# Synaptic Structure and Function in the Mouse Somatosensory Cortex during Chronic Pain: *In Vivo* Two-Photon Imaging

**DOI:** 10.1155/2012/640259

**Published:** 2012-02-22

**Authors:** Sun Kwang Kim, Kei Eto, Junichi Nabekura

**Affiliations:** ^1^Division of Homeostatic Development, National Institute for Physiological Sciences, Okazaki, Aichi 444-8585, Japan; ^2^Department of Physiology, College of Oriental Medicine, Kyung Hee University, Seoul 130-701, Republic of Korea; ^3^Department of Physiological Sciences, The Graduate School for Advanced Study, Hayama, Kanagawa 240-0193, Japan; ^4^Core Research for Evolutional Science and Technology, Japan Science and Technology Agency, Kawaguchi, Saitama 332-0012, Japan

## Abstract

Recent advances in two-photon microscopy and fluorescence labeling techniques have enabled us to directly see the structural and functional changes in neurons and glia, and even at synapses, in the brain of living animals. Long-term *in vivo* two-photon imaging studies have shown that some postsynaptic dendritic spines in the adult cortex are rapidly eliminated or newly generated, in response to altered sensory input or synaptic activity, resulting in experience/activity-dependent rewiring of neuronal circuits. *In vivo* Ca^2+^ imaging studies have revealed the distinct, input-specific response patterns of excitatory neurons in the brain. These updated *in vivo* approaches are just beginning to be used for the study of pathophysiological mechanisms of chronic diseases. In this paper, we introduce recent *in vivo* two-photon imaging studies demonstrating how plastic changes in synaptic structure and function of the mouse somatosensory cortex, following peripheral injury, contribute to chronic pain conditions, like neuropathic and inflammatory pain.

## 1. Introduction

Chronic pain initiated by tissue or nerve injury is a major challenge to clinical practice as well as basic neuroscience [1]. Peripheral neuropathic or inflammatory injury triggers structural and functional plastic changes in the cortical pain neuromatrix including the primary somatosensory cortex (S1) and anterior cingulate cortex (ACC), which results in altered nociceptive signal processing, such as mechanical allodynia (painful response to innocuous mechanical stimuli) [2, 3]. In previous brain imaging studies, for example, patients and animals under chronic neuropathic or inflammatory pain states showed increased activation and somatotopic reorganization in the S1, the extent of which was highly correlated with the pain intensity levels [4, 5]. Changes in gray matter density and in cortical thickness of the pain-related areas including the S1, ACC, and insula cortex were also found in chronic pain subjects [6, 7]. Further, several strategies to reduce the S1 hyperexcitation and reorganization showed benefits against chronic pain [8–11]. Although much is now known about such macroscopic changes in the cortex, it remains to be elucidated how and to what extent cortical connections are remodeled during chronic pain, and how such remodeling affects pain behaviors. This paper focuses on the recent findings from *in vivo* two-photon imaging studies to address the aforementioned questions: (1) the rapid and phase-specific remodeling of synaptic structures in the S1 of neuropathic pain mice following peripheral nerve injury [12] and (2) the enhanced activity of the S1 neurons affecting ACC neuronal function during inflammatory pain [13].

## 2. Structural Remodeling of Synapses in the Mouse S1 during Neuropathic Pain

Based on static measurements between different groups and on macroscopic observations, it has been believed that structural rewiring of neuronal connections in the cortex during chronic pain following injury takes much longer periods of time (i.e., months or years) than the development of allodynia and functional changes in cortical excitation, such as long-term potentiation (LTP), that occur within days or weeks [3, 14]. Recent long-term *in vivo* two-photon imaging studies have revealed that novel sensory experiences, or motor learning, can however induce rapid structural reorganization of synaptic connections in the related sensory or motor cortex that occur within days and are temporally correlated with functional plasticity of cortical circuits [15–19]. Given the high similarity of the mechanisms between chronic pain and learning and memory, as exemplified by the two forms of use-dependent synaptic plasticity “central sensitization” and “LTP”, respectively, [3, 20–22], it seemed reasonable to hypothesize that neuronal circuits in the S1 of intact brain would be remodeled following peripheral nerve injury with a similar time scale of the development of neuropathic pain behaviors and S1 hyperexcitability. Supporting this idea, several *in vitro* studies using intracellular filing of neurons in rat brain slices with biocytin suggested that dendritic structures in the S1 and medial prefrontal cortex were significantly changed at one or two weeks after peripheral nerve injury [23, 24]. A recent long-term *in vivo* two-photon imaging approach [12], described below, has now shown that in living mice structural changes in cortical circuits can indeed occur within the same rapid time scale as functional changes, indicating that the previous notion about only slow and chronic changes in cortical connections occurring in chronic pain states should be modified.

### 2.1. Time Course of the Development of Mechanical Allodynia and the S1 Hyperexcitability following Neuropathic Injury

**Figure 1:**
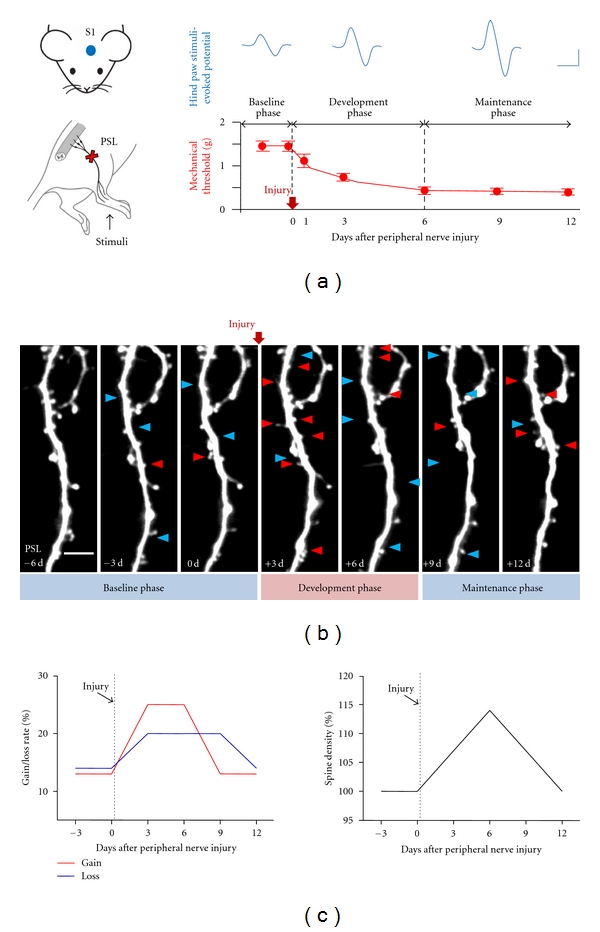
Rapid and phase-specific structural plasticity of dendritic spines in the S1 following peripheral nerve injury. (a) Left panels: schematic diagram of the PSL injury model and associated investigations in the S1. Bottom graph outlines the development and maintenance phases of mechanical allodynia following PSL injury with the upper panels showing the concurrent phase-dependent increases in somatosensory-evoked potentials in the S1. Scale bars, 50 ms (horizontal) and 50 *μ*V (vertical). (b) *In vivo* two-photon time-lapse images of the same S1 dendritic segment following PSL injury. Arrowheads indicate the spines that generated (red) and eliminated (blue) when compared with the previous imaging session. Scale bar, 5 *μ*m. (c) Schematic representation of the time course of changes in spine gain/loss rates (left) and overall spine density (right) during neuropathic pain. (a–c) Reproduced and adapted, with permission, from [12].

Neuropathic pain following partial sciatic nerve ligation (PSL) is a well-characterized mouse model [25, 26] that can be subdivided, based on the behavioral signs of mechanical allodynia, into an early “development” phase (~post-PSL to day 6) and a later “maintenance” phase (day 6 onwards) (Figure 1(a)). Hind paw stimulation-evoked cortical field potentials recorded in the S1 layer 1 *in vivo *[27] significantly increase in the development phase, and to an even greater extent in the maintenance phase (Figure 1(a)). From these behavioral and electrophysiological results, it might be expected that spine turnover in the S1 of neuropathic mice might be enhanced in a phase-dependent manner as well. To test this prediction, we utilized a transgenic mouse that sparsely expresses enhanced green fluorescent protein (GFP-M line) in cortical neurons [28] and set about to repeatedly image with the two-photon laser scanning microscope the same apical dendrites of functionally identified adult S1 hind-paw layer 5 pyramidal neurons, before and after PSL injury (Figure 1(b)). Layer 5 pyramidal neurons are the major output cells in the S1 and their distal tuft dendrites that are innervated by thalamocortical and corticocortical long-range projections as well as local circuit inputs encode information about somatosensory stimuli [29]. However, some consideration had to first be given about the most appropriate imaging procedure.

### 2.2. Chronic Cranial Window for Long-Term *In Vivo* Two-Photon Imaging during Chronic Pain

For long-term high-resolution imaging of synaptic structures in the cortex of living adult mice, the overlying opaque skull bone should be partially removed to make a cranial window. There are broadly two types of cranial window, namely, the “thinned-skull” window and the “open-skull” glass window [30–32]. The thinned-skull preparation is achieved by thinning the skull bone over a small area (about 1 mm in diameter) to be less than 30 *μ*m thick, whereas in the open-skull preparation a piece of the cranial bone is removed (about 2–5 mm in diameter), leaving intact the dura, and the exposed brain is covered with a thin glass coverslip (*for detailed methods and their pros and cons, see protocol articles,* [33–36]). Although thinned-skull preparation has many advantages (e.g., less invasive), it is difficult to image the same area more than 4 times, and rethinning procedure is required every imaging session, which is not necessary in the open-skull preparation. However, the mechanical sensitivity of the hind paw moderately increased for 2 weeks after an open-skull glass window implantation procedure, before completely returning to normal at 4 weeks after implantation [12]. Thus, long-term *in vivo* imaging experiments during neuropathic pain could only commence from 1 month after the cranial window implantation.

### 2.3. Dendritic Spine Dynamics Strikingly Increased during the Development Phase of Neuropathic Pain, But Were Restored in the Maintenance Phase

High-magnification successful repeated imaging of individual dendritic spines (Figure 1(b)) revealed the unexpected result that there was a marked increase in spine turnover rate (*N*_gain_ + *N*_loss_/2*N*_total_), an excellent indicator of structural synaptic plasticity, during the development phase of neuropathic pain, but a turnover just rapidly decreased back to normal during the maintenance phase. The observed spine turnover changes in the PSL mice are region- and injury-specific, because little change was found in the barrel cortex of PSL mice and in the S1 hind paw area of sham control mice [12]. Considering the time-course of mechanical allodynia and S1 hyperexcitability together (Figure 1(a)), these spine turnover data may provide the structural and temporal correlates of neuropathic pain at the level of cortical synapse. It also suggests that neuropathic pain-specific formation of new connections and elimination of preexisting circuits occur mainly within the early phase of neuropathic pain. Even though large scale sprouting or retraction of axonal and dendritic arbors of pyramidal cells in the adult cortex of living animals is rarely seen in imaging over a few weeks [30, 37, 38], even after neuropathic injury [12], a minor fraction of persistent synapses added or subtracted by neuropathic injury or novel sensory experiences can sufficiently store specific long-term information [39, 40].

The rate of spine gain following PSL injury showed a striking increase during the development phase, together with relatively moderate increase in spine loss rate, resulting in significant increase in spine density at the end of the development phase (Figure 1(c)). Such an increase in spine density was mainly due to a significant upregulation of thin spines [41]. Interestingly, increased spine elimination following injury continued up to post-PSL 9 days, whereas the new spine formation rate was reduced to normal baseline levels from the beginning of the maintenance phase. As a result, spine density returned to control level on post-PSL 12 days (Figure 1(c)). Since the major fraction of new spines was transient in the S1 [12] and in other sensory cortex areas [17, 18, 42], irrespective of injury and novel experience, subsequent elimination of new spines that had been generated during the development phase contributes to the above result, reflecting the refinement process of new connections.

### 2.4. Early Afferent Hyperactivity Is the Main Cause of Mechanical Allodynia and of S1 Synapse Remodeling

Preemptive or perioperative analgesia is based on the “pain memory” concept, in which an injury-induced afferent barrage can initiate the development of subsequent sensitization in the central nervous system that in turn contributes to the persistence of chronic pain [20–22, 43]. Analgesics and local nerve blockade before or during injury, but not after, can prevent or reduce pain, analgesic requirements, and abnormal changes in the spinal dorsal horn [44–46]. Similarly, the development of mechanical allodynia and upregulation of spine turnover following nerve injury were completely inhibited by local blockade of afferent activity in the injured sciatic nerve throughout the development phase [12]. However, the same nerve blockade, if begun in the maintenance phase, showed only a transient and moderate reduction in allodynia [12, 44]. These findings not only suggest the important role of early afferent hyperactivity-induced remodeling of the S1 synaptic structures in maintaining neuropathic pain, but also extend the pain memory hypothesis to the individual synapse level in the cortex.

### 2.5. Neuropathic Injury-Specific Formation of New Persistent Spines and Elimination of Preexisting Spines

**Figure 2:**
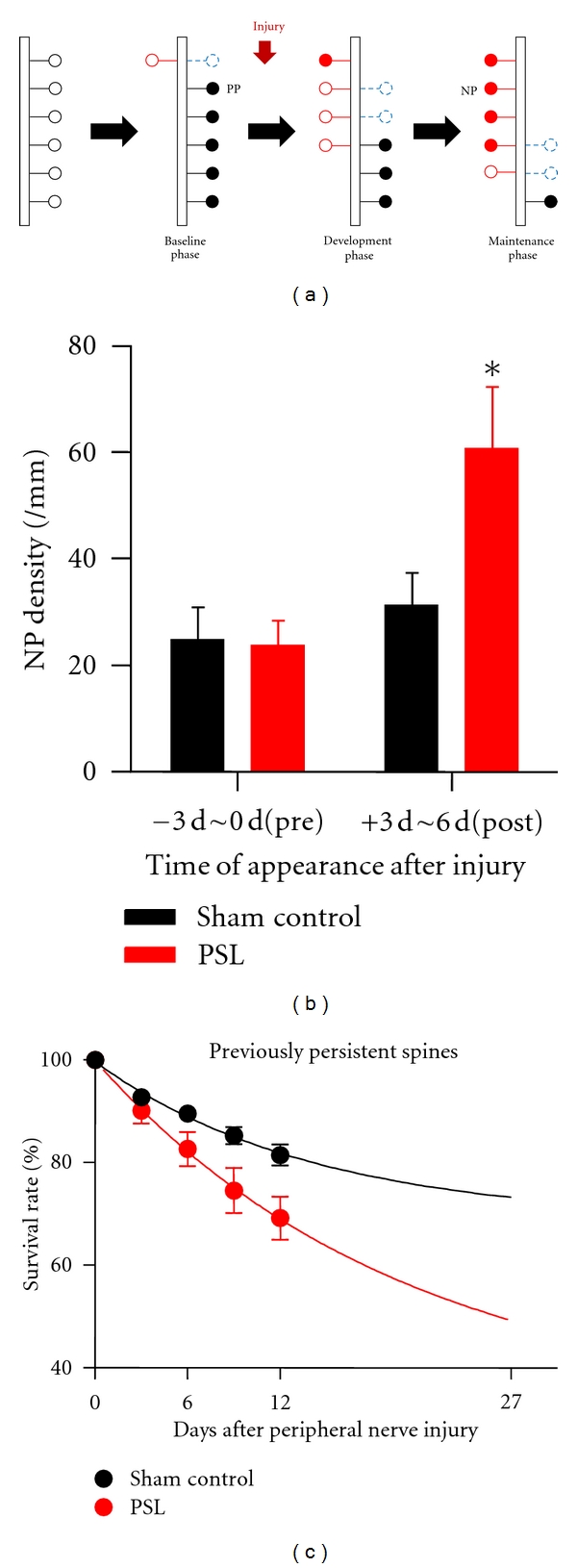
Neuropathic pain injury increases the formation of new persistent spines and increases elimination of previously persistent spines. (a) Simplified model for spine formation (red open circles) and elimination (blue, dashed open circles) under basal conditions and during neuropathic pain. PP: previously persistent spines (black filled circles). NP: new persistent spines (red filled circles). Note the increase in NP spines (b) and decrease in PP spines (c) following PSL injury. (b and c) Reproduced and adapted, with permission, from [12].

The increased number of new persistent spines that are generated during sensory manipulation or motor training has been considered as representing long-term memory traces [47]. Monocular deprivation [17], motor learning [19], and partial whisker trimming [42], or an enriched environment for whisker stimulation [18], all increased the number of new persistent (NP) spines on layer 5 pyramidal cells in the mouse visual, motor and barrel cortex, respectively. Consistent with those findings, the number of NP spines that appeared during the development phase of neuropathic pain was significantly higher than that of NP spines that appeared both before PSL, and in time-matched control groups (Figures 2(a) and 2(b)). Notably, the volume of NP spines that appeared during the development phase of neuropathic pain was substantially increased in the maintenance phase [12]. Since the spine volume is positively correlated with synaptic strength [40, 48], this result, together with increased number of NP spines (Figures 2(a) and 2(b)), probably indicates the encoding and subsequent enhancement of a neuropathic pain memory at single synapses, underlying the long-lasting nature of neuropathic pain.

Although sensory manipulation or motor learning upregulates NP spines in the relevant cortical area [12, 17–19, 42] as mentioned above, such manipulations did not change the final overall spine density with one exception [17], perhaps reflecting a limitation of the brain's capacity to accumulate all NP and previously persistent spines in response to each new experience or each new incident of learning. Thus, cortical circuit rewiring requires the removal of unnecessary preexisting connections at the same time as NP synapse formation. Indeed, a significant proportion of previously persistent spines were selectively eliminated over 2 weeks following PSL injury (Figures 2(a) and 2(c)) and simple extrapolation of those results with a single exponential fit estimated that 2/3rds of previously persistent spines in PSL mice might be completely eliminated [12]. This suggests a significant impact of neuropathic injury on cortical circuits throughout the whole life of chronic pain subjects.

## 3. Functional Plasticity of Intra- and Interregional Cortical Circuits during Persistent Inflammatory Pain

As mentioned above, peripheral injury induces functional plastic changes in the cortical pain neuromatrix including the S1 and ACC, where the integration and processing of pain signals might occur. Although it has been suggested that the S1 and ACC play a major role in the sensory and emotional aspects of pain, respectively [7, 49, 50], little is known about if and how the two cortical regions interact with each other under chronic pain conditions, and whether such interactions contribute to pain behaviors. Since layer 2/3 (L2/3) excitatory neurons in the S1 integrate sensory information originating from peripheral nerves via L4 neurons and transmit these signals to other pain-related cortical areas [51, 52], it would be a good strategy to determine the plastic changes in the S1 L2/3 neurons' function during chronic pain and then investigate how these changes may affect the ACC activity and pain behavior.

**Figure 3:**
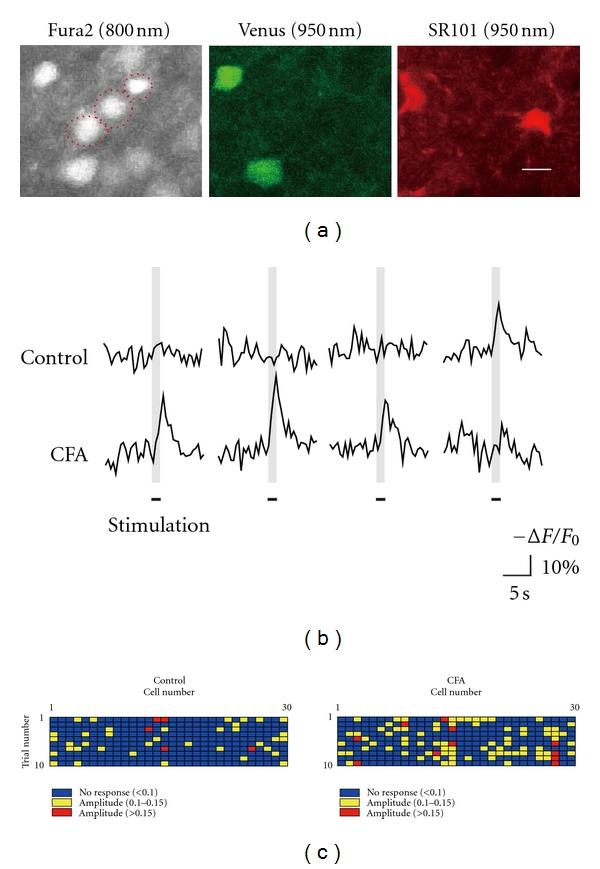
Enhanced activation of L2/3 excitatory neurons in response to hind paw stimulation and in response to stimulation of L4 neurons. (a) Identification of the S1 L2/3 excitatory neurons (red dotted circles in the Fura2 image), inhibitory neurons (green, Venus image), and astrocytes (red, SR101 image). Ca^2+^ indicator (Fura2) was excited at 800 nm two-photon laser, whereas Venus and SR101 were excited at 950 nm laser. Only cells that were positive for Fura2, but not for Venus and SR101, (i.e., excitatory neurons) were included in analysis. Each subpanel shows the same imaging area of the mouse S1 L2/3. (b) Representative traces of Ca^2+^ transients in identified L2/3 excitatory neurons during the same intensity stimuli (mechanical stimuli of hind paw or electrical stimuli of L4 cells) under control conditions (top traces) and following CFA-induced inflammatory pain (lower traces). (c) Distribution of the amplitude of Ca^2+^ responses to 10 successive stimuli in a sample of 30 L2/3 excitatory neurons under control conditions (left) and following CFA-induced inflammatory pain (right). (a–c) Reproduced and adapted, with permission, from [13].

*In vivo* two-photon Ca^2+^ imaging in living transgenic animals expressing a fluorescence only in inhibitory neurons (Venus) [53], combined with a multicell bolus loading of Ca^2+^ indicators (fura-2) [54, 55] and the astrocyte-specific dye (Sulforhodamine 101, SR101) [56] allows us to distinguish the response of astrocytes, excitatory neurons, and inhibitory neurons (Figure 3(a)). Furthermore, the neuronal activity in tens or hundreds of each cell type can be monitored at the same time during peripheral sensory stimulation [54]. Such experiments, using the Complete-Freund's-Adjuvant-(CFA-) induced inflammatory pain model in mice, showed that the probability and amplitude of Ca^2+^ transients in the S1 L2/3 excitatory neurons, and the number of cells activated by either low-intensity hind paw stimulation or electrical stimulation of the L4 region, are significantly increased (Figures 3(b) and 3(c)). Considering that the amplitude of evoked Ca^2+^ transients reflects the number of action potentials [55], these results suggest that the excitability of the S1 L2/3 neurons in response to mechanical stimulation of the hind paw is enhanced under inflammatory pain condition, at least in part through an amplified synaptic transmission from L4. 

Since the experience of pain is related to activation of both sensory and emotional aspects, which are thought to be differentially processed in the S1 and ACC, respectively [49, 50], the two cortical areas are expected to interact with each other [57]. Pharmacological inhibition of the S1 L2/3 neuronal activity in CFA-injected mice, but not in normal control mice, significantly attenuated the ACC activity evoked by hind paw stimulation, as well as significantly attenuating allodynia [13]. Conversely, pharmacological activation of the S1 L2/3 enhanced the ACC activity and induced an allodynic behavior in normal mice [13]. Therefore, there are considerable interactions between the S1 and ACC when the S1 L2/3 excitatory synaptic transmission is abnormally enhanced, which contribute to chronic pain behavior.

## 4. Concluding Remarks

**Figure 4:**
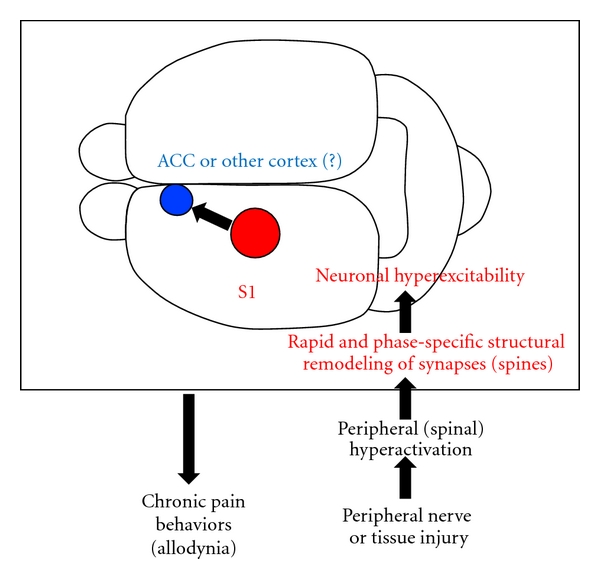
Working hypothesis for the cortical mechanism of peripheral injury-induced chronic pain. We propose that peripheral injury (nerve ligation or inflammation) induces rapid structural and function remodeling of S1 cortical synapses as described in the text. This, alongside possible other contributions of inhibitory interneurons and glia, results in hyperexcitability of excitatory S1 cortical neurons. These may project and interact with other regions within the pain “neuromatrix”, such as the ACC, to result in chronic pain behaviors such as allodynia.

In conclusion, we propose the following working hypothesis of the cortical mechanisms of chronic pain (Figure 4): peripheral nerve or tissue injury induces peripheral hyperactivity [21, 58], which causes a rapid rewiring of S1 synaptic connections [12]. Such synaptic remodeling, including an increased synaptogenesis and synapse elimination, and an enhanced strength of persisting synapses, causes local hyperexcitability of S1 to peripheral stimulation and might also affect the ACC or other pain-related cortical areas, finally leading to chronic pain behavior (allodynia) [12, 13].

The applications of *in vivo* two-photon imaging to pain research, as described above, are still at an early stage. There remain many unsolved questions regarding the pathophysiological changes of cortical synaptic structures and neuronal functions during chronic pain. For example, what happens to cortical inhibitory neurons and their synapses during chronic pain? How do cortical glial cells, such as astrocytes and microglia, contribute to plastic changes in synaptic structure and function during peripheral injury-induced chronic pain? What is the causal relationship between chronic pain and cortical synaptic remodeling? How do several cortical and subcortical regions comprising the pain neuromatrix, including not only S1 and ACC, but also insular cortex and thalamus, interact with each other? We are optimistic that these and other important questions will be resolved in the near future.
